# Advanced (bio)fouling resistant surface modification of PTFE hollow-fiber membranes for water treatment

**DOI:** 10.1038/s41598-023-38764-9

**Published:** 2023-07-22

**Authors:** Hadi Taghavian, Miroslav Černík, Lukáš Dvořák

**Affiliations:** 1 Institute for Nanomaterials, Advanced Technologies and Innovation, Technical University of Liberec, Studentská 1402/2, 461 17 Liberec 1, Czech Republic; 2 Faculty of Mechatronics, Informatics and Interdisciplinary Studies, Technical University of Liberec, Studentská 2, 461 17 Liberec 1, Czech Republic

**Keywords:** Chemical biology, Biogeochemistry, Environmental sciences, Engineering

## Abstract

Membrane surface treatment to modify anti-(bio)fouling resistivity plays a key role in membrane technology. This paper reports on the successful use of air-stimulated surface polymerization of dopamine hydrochloride incorporated ZnO nanoparticles (ZnO NPs) for impeding the intrinsic hydrophobicity and low anti-(bio)fouling resistivity of polytetrafluoroethylene (PTFE) hollow-fiber membranes (HFMs). The study involved the use of pristine and polydopamine (Pdopa) coated PTFE HFMs, both with and without the presence of an air supply and added ZnO NPs. Zeta potential measurements were performed to evaluate the dispersion stability of ZnO NPs prior to immobilization, while morphological characterization and time-dependency of the Pdopa growth layer were illustrated through scanning electron microscopy. Pdopa surface polymerization and ZnO NPs immobilization were confirmed using FT-IR and EDX spectroscopy. Transformation of the PTFE HFM surface features to superhydrophilic was demonstrated through water contact angle analysis and the stability of immobilized ZnO NPs assessed by ICP analysis. Anti-fouling criteria and (bio)fouling resistivity performance of the surface-modified membranes were assessed through flux recovery determination of bovine serum albumin in dead-end filtration as well as dynamic-contact-condition microbial evaluation against *Staphylococcus* spp. and *Escherichia coli*, respectively. The filtration recovery ratio and antimicrobial results suggested promising surface modification impacts on the anti-fouling properties of PTFE HFM. As such, the method represents the first successful use of air-stimulated Pdopa coating incorporating ZnO NPs to induce superhydrophilic PTFE HFM surface modification. Such a method can be extended to the other membranes associated with water treatment processes.

## Introduction

Membranes are commonly used in water treatment systems due to their small footprint, reasonable operating costs, high selective separation efficiency, and high quality of final permeate^1–3^. Membrane filtration is a versatile technology that can be coupled with other water separation systems, including micro- and ultra-filtration in bioreactors, where it represents an autonomous substitute for secondary clarifiers and nanofiltration in the treatment of drinking water^4,5^. An important obstacle impeding the development of membranes as the primary option in water treatment systems, however, is (bio)fouling^4,6^.

Membrane fouling is the most common issue reducing membrane productivity during filtration^7^. Attachment of inorganic matter, deposition of organic residuals, entrapment of particulate substrates, and accumulation of microorganisms can all create a cake layer on the membrane surface, leading to pore blockage, increased transmembrane pressure, increased energy consumption, a reduction in permeate flux, and inefficient membrane functionality^4,7^. By integrating practical results of membrane ultrafiltration in bioreactor systems with theoretical models of cake layer blockage, Yang et al.^8^ concluded that such cake layers were the main foulant of membranes, with Wardani et al.^9^ noting that the actual rate of fouling was the result of a correlation between the membrane’s intrinsic properties and foulant composition.

Due to ample physical and chemical stability, synthetic organic polymers are increasingly being used as raw materials for the production of ultrafiltration hollow fiber membranes (HFMs)^9,10^. For instance, polytetrafluoroethylene (PTFE) is often used due to its comparatively higher inherent physiochemical properties than other conventional HFMs applied for water treatment. PTFE has high chemical resistivity allowing its use in various water treatment processes, even when exposed to aggressive or corrosive substances. PTFE also exhibits high temperature tolerance which helps to withstand at elevated temperatures without compromising its physical integrity; and flexibility which enables the submerged membranes to easily shake and move within bioreactor system without the fear of tearing^11^. From these reasons, PTFE has been chosen in this study. Nevertheless, PTFE HFMs have hydrophobic characteristics that facilitate the adsorption of proteins, fatty acids, and most filamentous microorganisms (MO) in a process that contributes to membrane fouling^11,12^. The dominant phase of these foulant groups is hydrophobic, meaning that it is attracted to the organic membrane’s hydrophobic surface^13–15^, thereby increasing the fouling rate through attachment to the membrane surface or by becoming trapped inside the membrane pores^12^.

Surface modification of membranes is now a popular technique for producing fouling-resistant, high-functioning membranes in wastewater treatment systems^11,12^. Recently, several studies have investigated the hypothesis that hydrophilic surface-modified membranes are less susceptible to fouling than standard hydrophobic membranes^16^. Galiano et al.^17^, for example, applied a polymerized surfactant-containing coating to a polyethersulfone (PES) membrane surface to increase its hydrophilicity and fouling resistivity against organic textile dyes in a bioreactor. The results demonstrated that the modified membrane facilitated the maintenance of 65% of its initial permeability, representing a significant 43% improvement compared to the unmodified membrane, which only exhibited a 22% recovery^17^. In a follow-on study, Johnson et al.^18^ confirmed that hydrophobic membranes used in wastewater treatment displayed high adhesion properties that increased the likelihood of fouling. The results indicated that the adhesion forces between the hydrophilized membranes and the measuring probe within the model textile dye wastewater were lower than 0.5 mN m^−1^ in the best samples, whereas the unmodified PES membrane exhibited an adhesion force of 1.5 mN m^−1^^18^. Accordingly, hydrophilic surface treatment of HFMs has become an essential modification approach, particularly for hydraulic systems containing significant levels of hydrophobic particulate matter.

Recently, a new surface modification technique based on mussel-inspired polydopamine (Pdopa) has been explored as a simple method for hydrophilizing many different polymer membrane surfaces^19^. The Pdopa coating easily attaches to membrane surfaces, after which, under suitable pH and ambient conditions, dopamine hydrochloride undergoes a process of oxidative polymerization^9^. Not only is this a bio-inspired technique, which is of increasing importance in drinking water and wastewater treatment^19^, the unique properties of the Pdopa coating make it of special interest for the surface modification of various specialized membranes^19,20^. An et al.^12^, for example, used Pdopa with cysteine to modify the surface of polyvinylidene fluoride (PVDF) HFMs to increase anti-protein adsorption as high as 30% improvement to be around 70 μg cm^−2^ bovine serum albumin (BSA) adsorption in comparison with the pristine one with 100 μg cm^−2^. Likewise, Yang et al. reported promising results for Pdopa coating on the functioning and anti-fouling properties of osmosis membranes with only 3.69% water flux reduction during performance (in comparison with 20.9% water flux reduction for pristine membrane) and flux recovery ratio (FRR) higher than 80%^20^. Similarly, da Silva et al.^21^, reported functional improvement of PVDF membranes after applying Pdopa and TiO_2_ (1%) to increase the water permeability from 462 L m^−2^ h^−1^ bar^−1^, for pristine one to 1063 L m^−2^ h^−1^ bar^−1^. Bonyadi et al.^22^, increased the recovery ratio of PES membranes by applying Pdopa-incorporated functionalized SiO_2_ from 29.8 up to 97.5% due to the supreme hydrophilicity. Finally, Pakizeh et al.^23^ used Pdopa and TiO_2_ nanoparticles (NPs) to improve the FRR of polyphenylsulfone membranes. They achieved up to 87% FRR in comparison with 60.2% for pristine membrane during removal of Direct Orange102 dye from aqueous solutions mainly due to the improvement in hydrophilicity and separation properties^23^. Kim et al.^24^ suggested that the catechol and amine groups in the Pdopa structure allow it to be used as a facile technique for covalently immobilizing NPs and other coatings on a membrane’s surface. Furthermore, Park et al.^25^ used Pdopa coating as a bio-inspired “glue” for attaching a vanillin coating to the surface of a feed spacer to modify its biofouling resistivity, while Liu et al.^19^ used Pdopa to immobilize a zwitterionic copolymer on the surface of a PVDF membrane to improve its permeability and fouling resistivity. Finally, many studies have reported that Pdopa coatings increase the antimicrobial properties of the substrate to which it is applied^26,27^.

Owing to the existence of four fluorine elements, PTFE monomers tend to have high surface tension, making them resistant to most of the surface modifications outlined above. However, the Pdopa molecule contains catechol and amine groups, enabling it to attach to nearly any nearby surface. PTFE, however, has low surface energy, meaning that Pdopa growth on the outer surface tends to be less effective. To address this, it has been proposed the use of an air-stimulation method to strengthen oxidation of dopamine hydrochloride and polymerization of the Pdopa coating, with Wardani et al.^9^, for example, reporting beneficial results after using air-assistance for hydrophilic surface modification of polypropylene membranes. Consequently, application of a similar air-stimulated, bio-inspired technique may be a promising solution for surface modification of PTFE HFMs. However, while Pdopa surface modification could mitigate the attraction of biofoulants, there is still a need for an effective antimicrobial treatment to address bacterial growth and biofilm formation on membrane surfaces. A potential solution to this problem is the immobilization of inorganic particles with antimicrobial properties onto the membrane’s surface; indeed, a number of recent studies have reported the method to be both effective and long-lasting^23,28–30^.

Of the various inorganic particles proposed for anti-(bio)fouling modification of membranes, surface treatment using zinc oxide (ZnO) NPs has been particularly successful at reducing microorganism growth, with cytotoxicity assessments on inorganic materials confirming that ZnO NPs display low overall toxicity and high selectivity for combating bacteria, while simultaneously remaining non-toxic to cells^31–33^. Utilization of ZnO NPs during Pdopa surface modification of PTFE HFMs, therefore, is likely to prove effective in recovering initial flux in water treatment while also providing long-lasting biofouling resistivity.

While some modification methods based on use of inorganic NPs and hydrophilic surface modification have been reported as increasing the biofouling resistivity of membranes^24,34^, the results of the previous studies outlined above suggest that a methodology consisting of super hydrophilic surface modification of PTFE HFMs through hierarchical, air-stimulated Pdopa coating incorporating ZnO NPs could represent a promising new method for modulating membrane biofouling. The following study, therefore, outlines a potential methodology for undertaking such a surface modification of PTFE HFMs using two simultaneous pathways, i.e. super hydrophilic surface modification and incorporation of ZnO NPs, and assessing its effectiveness as regards resistance to membrane biofouling.

## Material and methods

### Hollow fiber membrane modification

Oxidative polymerization of Pdopa on the PTFE HFM surface was carried out using an exclusive air-stimulated procedure, which demonstrated the impact of dissolved oxygen on the auto-oxidation of Pdopa^35^. Initially, PTFE HFMs (Dongyang Hanchen Membrane Technology Co. Ltd., China) of identical lengths were used to prepare a series of testing modules by packing the HFMs into a tube and connecting this to an air compressor. A laboratory stand equipped with a clamp was then used to immerse the membrane module into a reactive solution comprising 2.0 mg mL^−1^ of dopamine hydrochloride (99%, MW = 189.84 g mol^−1^, Alfa Aesar, USA) dissolved in a pH adjusted (pH = 8.5) 10 mmol L^−1^ solution of TRIS buffer (Tris(hydroxymethyl)aminomethane, 99.5%, MW = 121.14 g mol^−1^, Penta) in deionized (DI) water. The supplied air was utilized to stimulate the oxidation polymerization of dopamine hydrochloride monomers, thereby promoting the growth of a Pdopa coating on the surface of PTFE HFMs. The complex reactive solutions were stirred overnight and continued for up to 28 h. During this time, the Pdopa layer started to grow spontaneously on the membrane surface, with its thickness depending on time. The increase in Pdopa deposition on the membrane surface was monitored visually by a gradual color change from colorless to black. After the chosen time of 4, 14, and 24 h (Table 1), which was determined based on the surface characteristic conversion, the membrane module was removed from the reactive solution and gently washed with DI water to remove unreacted agents and then soaked in DI water.

**Table 1:** Overview of samples tested in this study.

Sample name	Modification	Pdopa polymerization time (h)	ZnO (mg mL^−1^) in EtOH
Blank	Pristine PTFE HFM	0	0
Pdopa 4	Pdopa4@PTFE HFM	4	0
Pdopa 14	Pdopa14@PTFE HFM	14	0
Pdopa 24	Pdopa24@PTFE HFM	24	0
Sample 1	ZnO 0.5 & Pdopa 24@PTFE HFM	24	0.5
Sample 2	ZnO 1 & Pdopa 24@PTFE HFM	24	1
Sample 3	ZnO 1.5 & Pdopa 24@PTFE HFM	24	1.5

**Figure 1:**
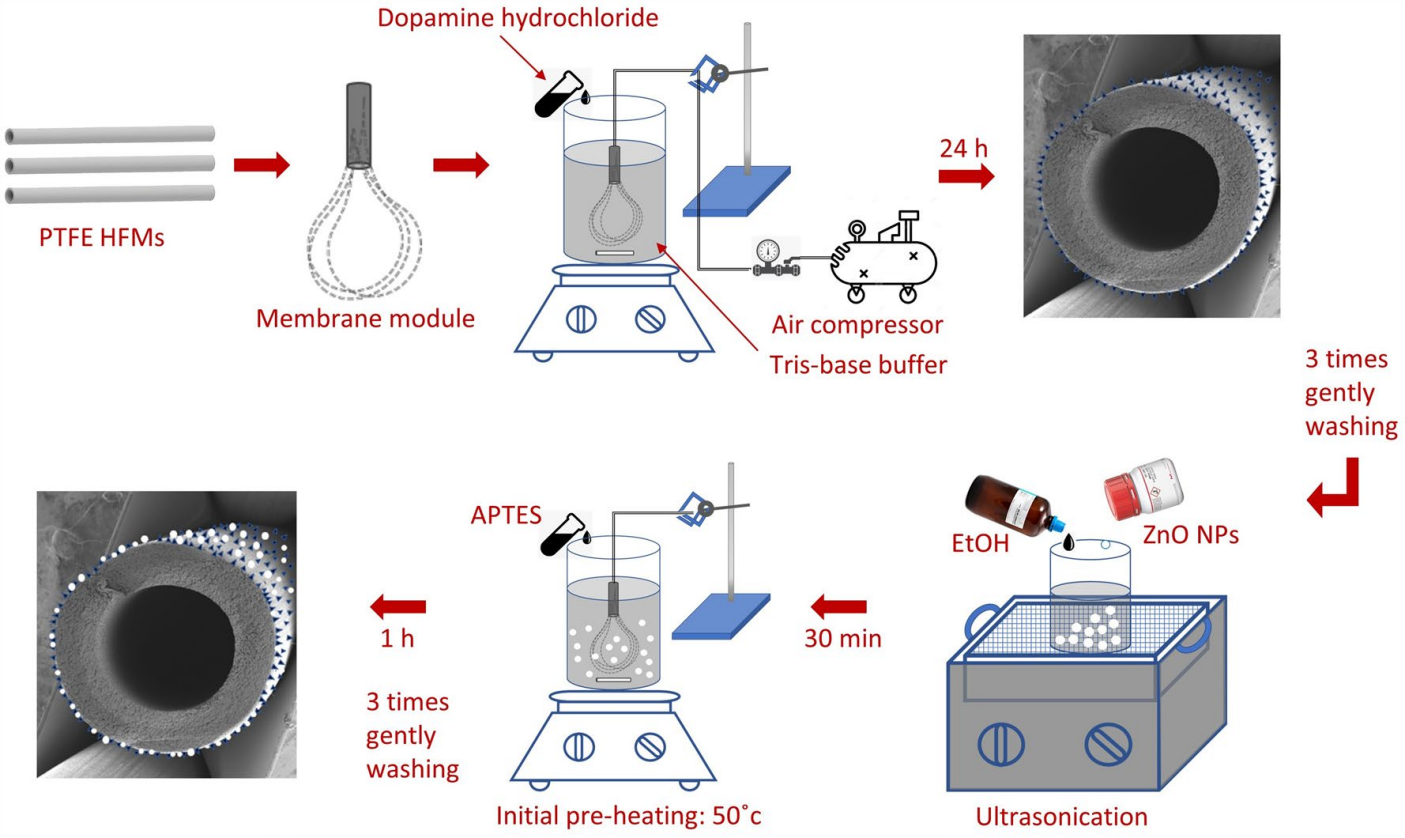
Hierarchical steps for surface modification via air-stimulated Pdopa surface polymerization and ZnO NPs immobilization.

In the second modification stage, ZnO NPs (~ 80%, MW = 81.39 g mol^−1^, Sigma-Aldrich, USA) were immobilized onto the surface of the Pdopa-coated PTFE HFMs. ZnO NPs were first dispersed in ethanol (EtOH, 96%, MW = 46,07 g mol^−1^, Penta) at concentrations of 0.5, 1.0, and 1.5 mg mL^−1^ (Table 1), vortexed, and then ultrasonicated (Bandelin, Sonorex digitec, Germany; 35 kHz) for 30 min at laboratory temperature. At this point, the zeta potential of the mixtures was evaluated to ensure sustainable dispersion of the nanoparticles (see below). Next, (3-Aminopropyl)riethoxysilane (APTES, 98%, TCI, USA) was added to the mixture at 2% v/v and then vigorously vortexed. The initial temperature of the complex solution was adjusted to 50 °C, following which the membrane modules were quickly immersed in the complex solution and left for one hour. Subsequently, the membrane modules were taken out and washed three times to ensure the complete removal of all non-reactive agents. Figure 1 shows a schematic illustration of the hierarchical steps of surface modification.

**Figure 2:**
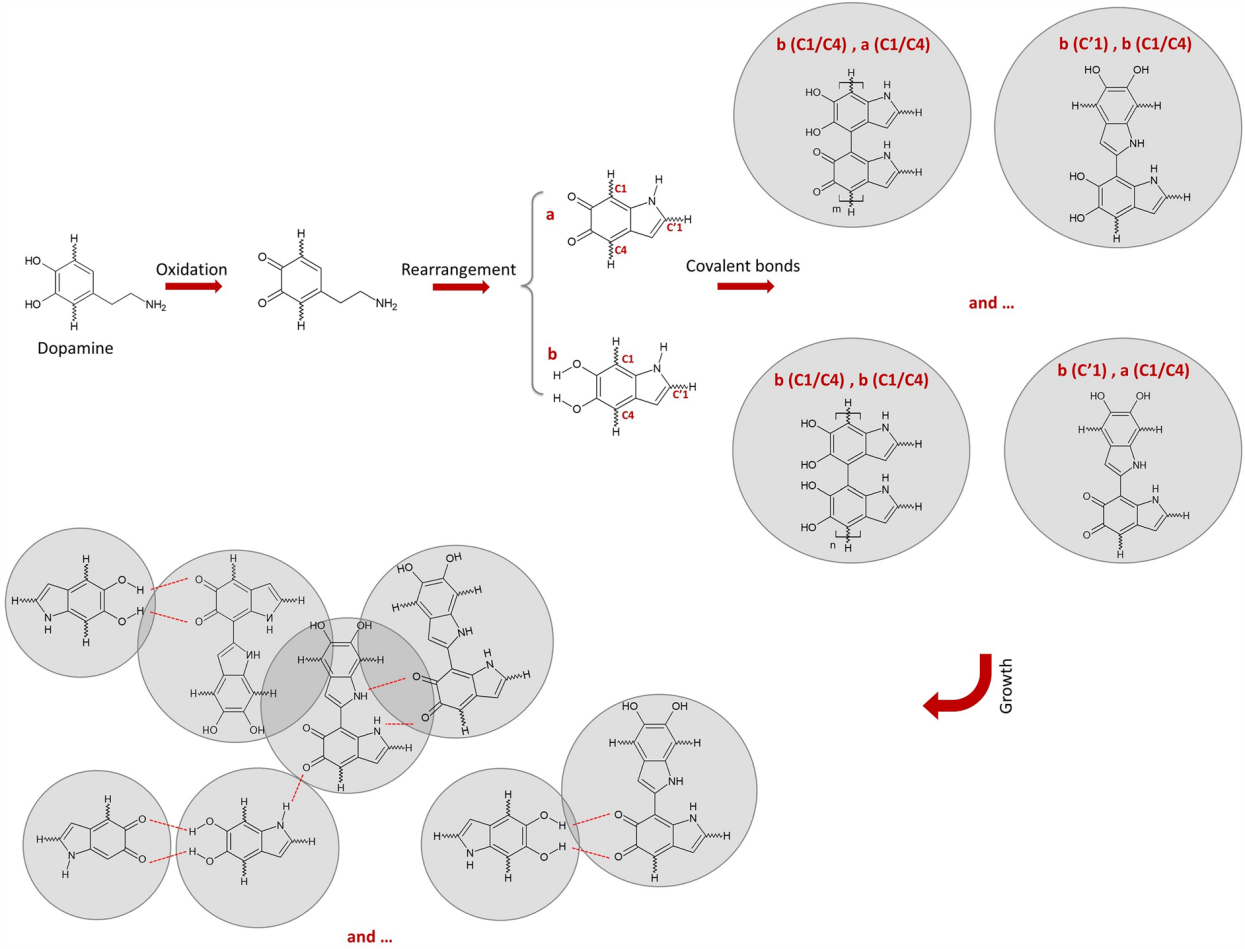
Schematic chemical process illustrating the polymerization of dopamine hydrochloride from Pdopa.

To summarize, the PTFE HFM modules were immersed in a pH-adjusted (pH = 8.5) TRIS buffer and dopamine hydrochloride solution while stirring continuously at 100 rpm on the magnetic stirrer. A Pdopa coating then grew gradually on the PTFE HFM, followed by oxidation, rearrangement, and polymerization of dopamine hydrochloride (see Fig. 2 for a schematic illustration of the chemical processes involved). In effect, dopamine hydrochloride monomers are oxidized in the alkaline condition, after which they lose hydrogen ions from hydroxide groups to form radicals. After rearrangement to a 5,6-dihydroxyindoline (DHI) structure, covalent bonds are formed from, the carbon one (C1) and carbon four (C4) of the DHI dopaquinone group and carbon one (C’1) of the DHI cyclopentane group^36,37^. In addition, there are π–π interactions between monomers attributable to the hydrogen bonding^36,37^. Covalent links and π-stacking hydrogen bonds between dopamine monomers create a layer of polydopamine in the form of NPs as polymerization increases^36,38,39^.

### Nanoparticle zeta potential and leaching test

The sustainability of the ZnO NPs dispersed in the EtOH carrier was evaluated for all samples by measuring the zeta potential in triplicate using a Zetasizer, nano series (Malvern instruments). Next, a NexION 300D inductive coupled plasma-optical emission spectrometer (ICP-OES; PerkinElmer, USA) with a detection limit of 1.0 μg L^−1^ was used to determine the stability of the modified coating by detecting the amount of ZnO NPs released from the membrane surface during filtration, using a modified leaching test^40,41^. First, the reservoir was filled with 5 L of DI water, following which the modified HFM module was fixed in a cell allowing dead-end filtration of DI water at transmembrane pressure of 1.0 bar. After filtration of 0.2 L (step 1), 0.5 L (step 2), 1.0 L (step 3), 1.5 L (step 4), 2.0 L (step 5), 2.5 L (step 6), 3.0 L (step 7), 3.5 L (step 8), 4.0 L (step 9), 4.5 L (step 10), and 5 L (step 11) of DI water, 10 mL of permeate was taken and 0.2 mL of HNO_3_ (14.58 mol L^−1^) added to ionize the Zn and increase detection accuracy. Afterwards, the membrane was unpacked from the module and dried at 105 °C for 2 h. The remaining ZnO NPs were entirely leached from the membrane surface by immersion in 60 mL of HNO_3_ (14.58 mol L^−1^) overnight at laboratory temperature. The amount of ZnO NPs released to the permeates and leached from the membrane surface diluted 5 × were evaluated by ICP-OES spectrometry. The stability of the ZnO NPs was calculated according to Eq. (1).

1 $${Zn}_{r} \left(\%\right)=\left(\frac{{({Zn}_{r})}_{i}}{\sum_{i=1}^{11}{({Zn}_{r})}_{i}+ {Zn}_{L}}\right)*100$$

where Zn_r_ is associated with the amount of ZnO NPs released at each water filtration interval volume, and Zn_L_ is associated with the ZnO NPs leached from the membrane surface.

### Membrane characterization

Morphological evaluations of the surface-treated PTFE HFMs were accomplished using scanning electron microscopy (SEM; TESCAN ORSAY HOLDING a.s, Czech Republic), while surface elemental analysis of the treated membranes was performed via energy-dispersive X‐ray (EDX) analysis. Fourier transform-infrared (FT-IR) spectrometer (Perkin Elmer, USA) was then used to study the chemical bonds of each sample. Surface hydrophilicity of the PTFE HFMs following surface modification was compared with a pristine membrane sample by measuring the water contact angle.

Average membrane porosity (%) was determined based on the weight of isopropyl alcohol (IPA) trapped in the membrane pores^9,42^. Samples of the modified and pristine PTFE HFMs were weighed (W_dry_), after which they were immersed in IPA and left for 24 h at laboratory temperature. Subsequently, they were removed and affixed vertically to drain the IPA. As soon as the solvent had evaporated from the percentage porosity (ε) was calculated according to Eq. (2)^9,42^.

2 $$\upvarepsilon \left(\%\right)= \frac{\left(\frac{{W}_{wet}-{W}_{dry}}{ {\uprho }_{IPA}}\right)}{\left(\frac{{W}_{wet}-{W}_{dry}}{{\uprho }_{IPA}}\right)+\left(\frac{{W}_{dry}}{{\uprho }_{PTFE}}\right)}*100$$

where ρIPA is the IPA density (0.78 g cm^−3^), and ρPTFE is the PTFE density (2.20 g cm^−3^).

### Membrane flux recovery

In this study, a dead-end cell connected to an air compressor (transmembrane pressure adjusted by an airflow meter) and water-supply tank (filled with DI water) was used to evaluate the flux recovery of PTFE HFMs before and after modification, with all filtration experiments taking place at laboratory temperature. The HFM modules were fixed into the cell, and the experiments began after 30 min, when the flux of each HFM had reached equilibrium at transmembrane pressure of 1.0 bar, and continued for 1 h. The flux (J) of each HFM after each filtration step, initial water flux (J_1_), BSA flux (J_BSA_), and secondary water flux (J_2_) was calculated according to Eq. (3)^10,23,43^. The membrane rejection ratio (RR) was measured according to Eq. (4) after filtration of BSA at a concentration of 1 mg mL^−1^ for a further 1 h^23,44,45^. Subsequently, the HFMs were ejected from the cell, washed with DI water, replaced in the cell, and refilled with DI water for evaluation of secondary water flux (J_2_) for a final 1 h and the percentage of flux recovery after protein filtration. The anti-fouling capability of the modified PTFE HFMs was evaluated by measuring FRR according to Eq. (5)^42,43^. The total membrane fouling percentage (R_total_) was evaluated according to Eq. (6)^42,46^. As some of the foulants may be removed by DI washing, these were considered reversible foulant (R_rev_) and were quantitatively calculated according to Eq. (7)^42,46^. Finally, the percentage of irreversible fouling (R_irr_), which cannot be removed, was calculated according to Eq. (8)^42,46^.

3 $$J=\frac{V}{\Delta t*A}$$

4 $$RR \left(\%\right)=\left(1- \frac{{C}_{permeate}}{{C}_{feed}} \right)*100$$

5 $$FRR \left(\%\right)=\left(\frac{{J}_{2}}{{J}_{1}}\right)*100$$

6 $${R}_{total}(\%)=\left(1- \frac{{J}_{BSA}}{{J}_{1}} \right)*100$$

7 $${R}_{rev}\left(\%\right)=(\frac{{J}_{2}- {J}_{BSA}}{{J}_{1}})*100$$

8 $${R}_{irr}\left(\%\right)=(\frac{{J}_{1}- {J}_{2}}{{J}_{1}})*100$$

where V is the permeate volume (L), A is the membrane surface area (cm^2^), Δt is filtration process time (h), C_permeate_ and C_feed_ are permeated, and initial BSA concentration, which were measured by the UV–vis spectroscopy analysis, respectively.

### Antimicrobial analysis

Antimicrobial effects of surface-treated PTFE HFMs with different amounts of immobilized ZnO NPs (sample 1, sample 2, and sample 3; according Table 1) were quantitatively compared under dynamic contact conditions according to standard ASTM E2149 by inoculation with gram-positive *Staphylococcus* spp. and gram-negative *E. coli*^47,48^. The dynamic contact condition test was chosen to eliminate problems emerging through the non-assurance of complete contact between the test samples and the bacterial inoculum. The gram-positive *Staphylococcus* spp. (CCM 2446) and gram-negative *E. coli* (CCM 7395) were obtained from the Czech Collection of Microorganisms (Brno, Czech Republic). The colony-forming units (CFU) of each bacterium were evaluated using the cultivation technique, whereby the bacterial stocks were cultured to obtain an exponential cell growth phase (approx. 1 × 10^5^ cells in 1 mL). Subsequently, the cells were cultivated, tenfold diluted up to 1000-fold dilutions in physiological solution (0.85% NaCl), then prepared for the membrane antimicrobial test on plate count agar (PCA) plates (Bio-Rad, France). To increase the accuracy, all analyses were duplicated at contact times of 0 and 24 h. CFUs were then evaluated after contact times of 0, 1, 3, 6, and 24 h to ascertain the antimicrobial activity of the samples over time.

A 0.5 g sample of each membrane was immersed in 25 mL of bacterial culture and shaken at 120 rpm in the laboratory temperature, after which 1.0 mL was taken from the diluted cell suspension and seeded onto the PCA plates after sample immersion at 0 h (i.e., directly after sample preparation), 1, 3, 6, and 24 h, the remaining samples being shaken throughout. Next, the samples were incubated at 37 °C for 48 h, following which all CFUs were determined at log_10_ (CFU mL^−1^). The reduction value (RV) and reduction percentage (R%) of the pristine and surface-modified PTFE HFMs was then evaluated based on the logarithmic value of antibacterial activity obtained, according to Eqs. (9) and (10), respectively^49,50^.

9 $${R}_{V}={log}_{10} {(B)}_{contact\, time}- {log}_{10} {(A)}_{contact\, time}$$

10 $$R (\%) =\left(\frac{B-A}{B}\right)*100$$

where B and A are CFUs of the blank and surface-modified samples, respectively.

## Results and discussion

### Hollow fiber membrane modification

**Figure 3:**
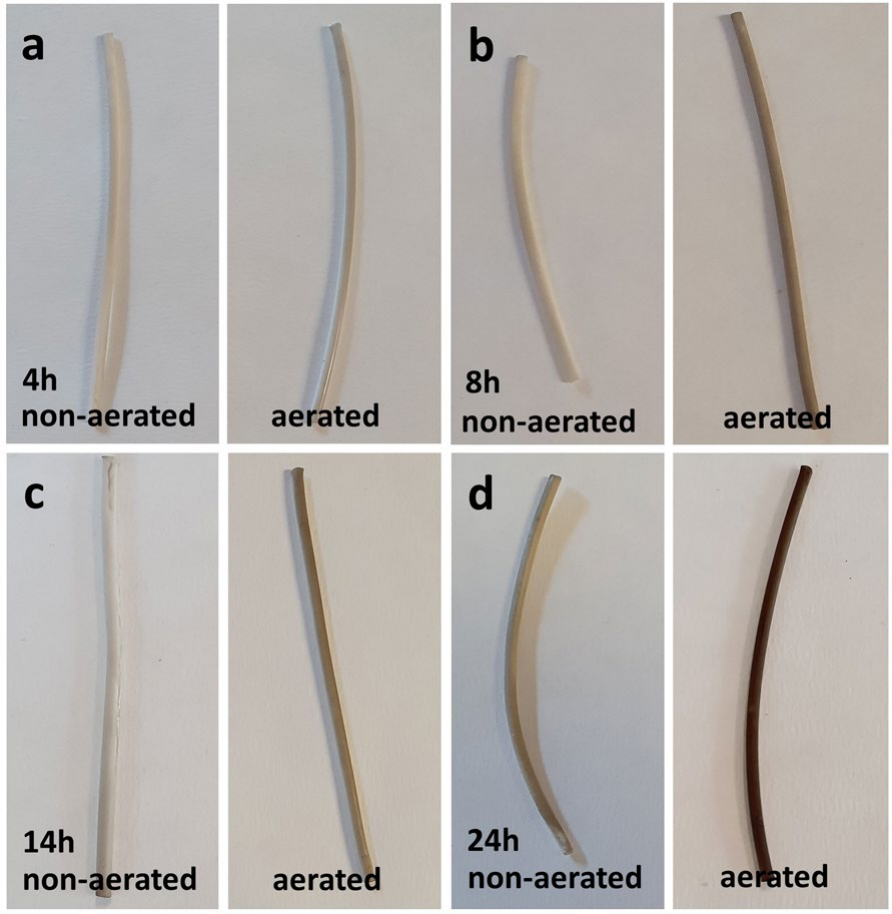
Comparison images of Pdopa coated PTFE HFMs under aerated and non-aerated polymerization conditions at different time intervals: (**a**) 4 h, (**b**) 8 h, (**c**) 14 h, and (**d**) 24 h.

In this study, the membrane modules were connected to an air compressor to stimulate oxide-activated self-polymerization of Pdopa. Visual observation confirmed that, in the absence of air, Pdopa failed to form an initial layer on the membrane surface, apparently due to the ultra-high hydrophobic properties of the PTFE. In comparison, there was a marked increase in the Pdopa growth layer on the PTFE HFM modules after 24 h of polymerization under aerated conditions (Fig. 3).

The polymerization time of Pdopa was incrementally increased in a stepwise manner until two key parameters were achieved: (1) high hydrophilicity and (2) high permeability. The findings demonstrated that prolonging time of Pdopa polymerization resulted in an augmentation of hydrophilicity. Upon reaching the 4 h, which acted as a threshold for attaining a water contact angle (WCA) below 90° and converting the surface from hydrophobic to hydrophilic, the membrane exhibited enhanced permeability. To further enhance hydrophilicity, the experiment was extended until it was observed that the surface became superhydrophilic after 24 h of oxidation polymerization, while still maintaining adequate permeability. However, extending the polymerization time beyond 24 h (up to 28 h) led to excessive growth of the Pdopa layer on the PTFE surface, no any additional effect on hydrophilicity was observed. Notably, as the Pdopa layer became thicker, the permeability decreased. Accordingly, it was revealed that 24 h aerated in set-up conditions Pdopa polymerization was the optimum processing time to form a uniform and homogeneous Pdopa coating on the PTFE HFMs, thereby creating fibers with a hydrophilic outer layer.

### Nanoparticles zeta potential

Table 2 shows the zeta potential of the samples distinguished by different ZnO content. Subsequently, a 0.5 mg mL^−1^ ZnO NP/EtOH solution (Sample 1; Table 2) displayed an average zeta potential of 17.97 mV, suggesting the solution was not properly dispersed. Solutions at 1.0 mg mL^−1^ (Sample 2) and 1.5 mg mL^−1^ (Sample 3), however, provided average zeta potentials of 35.03 and 32.2 mV, respectively (Table 2). As it is generally agreed that solutions with a zeta potential of ± 28 mV or higher display long-term sustainability due to their high dispersion levels^51,52^, reaction solutions of 1 mg mL^−1^ ZnO NP/EtOH would appear to contain ample dispersant for immobilization onto membrane surfaces.

**Table 2:** Zeta potential of different ZnO NP concentrations dispersed in ethanol.

Sample	Repeat	ZnO (mg mL^−1^)	Zeta potential (mV)	Ave. ZP (mV)	SD (mV)
Sample 1	1	0.5	18.8	17.97	1.1
	2		16.7		
	3		18.4		
Sample 2	1	1	38.7	35.03	4.0
	2		35.6		
	3		30.8		
Sample 3	1	1.5	33.6	32.20	1.2
	2		31.2		
	3		31.8		

### Membrane surface morphology and elemental composition

**Figure 4:**
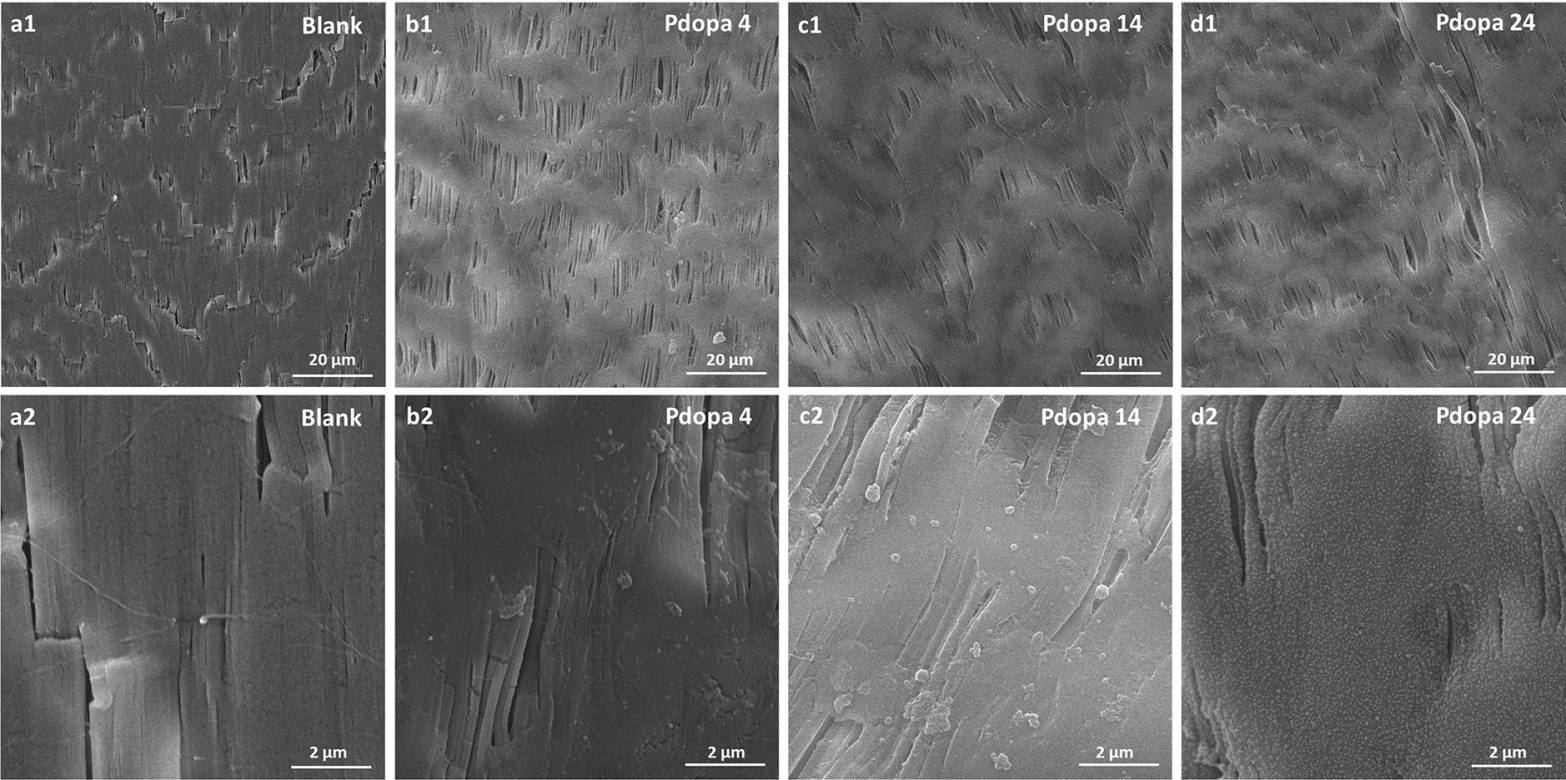
Scanning electron microscopy images of Pdopa-modified PTFE HFMs at different polymerization times and magnifications {2 kx = **a1**–**d1** & 20 kx = **a2**–**d2**}: (**a**) Pristine PTFE (blank), (**b**) Pdopa4@PTFE HFM (4 h), (**c**) Pdopa14@PTFE HFM (14 h), (**d**) Pdopa24@PTFE HFM (24 h).

SEM, which was used to monitor incremental growth of Pdopa on PTFE HFM surfaces at 4 h, 14 h, and 24 h after the start of polymerization, at higher magnification (20kx, Fig. 4a2–d2), revealed that increasing the polymerization time to 24 h resulted in uniform and homogeneous coverage of the whole PTFE HFM surface. At lower magnifications (2kx, Fig. 4a1–d1), the SEM images showed no pronounced difference in coverage at different polymerization times, suggesting non-dominant interference of Pdopa growth on the porous surface morphology.

**Figure 5:**
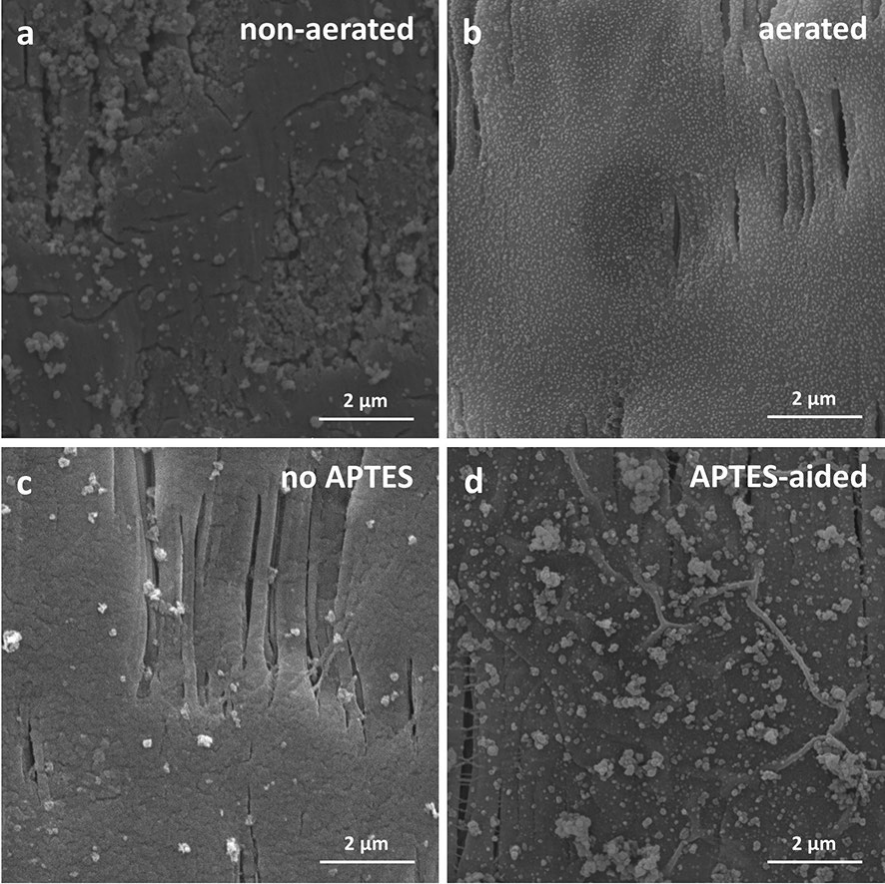
Scanning electron microscopy images and comparisons between different procedures: (**a**) non-aerated, (**b**) aerated Pdopa polymerization, (**c**) no APTES, and (**d**) APTES-aided ZnO NP immobilization on PTFE HFM.

The SEM images also highlighted the positive impact of air stimulation on Pdopa growth, with the Pdopa showing poor growth and a tendency to clump together on the PTFE surface rather than grow uniformly in the absence of aeration and diffused air in the reaction solution (Fig. 5a,b). Likewise, SEM images of with and without APTES-aided immobilized ZnO NPs on the membrane surface indicated that, unlike previous studies^21,23,25,26^, which highlighted the ability of Pdopa to hold NPs, the Pdopa coating does not contain the capability of robust immobilization of ZnO NPs on the surface of PTFE HFM (Fig. 5). While most of the ZnO NPs appeared to have been washed out from the surface following DI washing during the sample preparation process (Fig. 5c), use of APTES, a well-known crosslinker agent, appeared to reverse this process, allowing effective immobilization of ZnO NPs on the Pdopa-treated PTFE HFMs (Fig. 5d).

EDX analysis corroborated the SEM results, validating the minimal Zn immobilized by Pdopa on the membrane surface, while revealing significant immobilized Zn on the APTES-treated membranes. The level of Zn immobilization on the surface increased proportionally with increasing the concentration of ZnO NPs in the reaction mixture (Samples 1, 2, 3), as evidenced by the data presented in Table 3.

**Table 3:** Energy-dispersive X‐ray surface elemental analysis of samples 1 to 3.

	C		N		O		F		Si		Zn		Total
	Wt. (%)	At. (%)	Wt. (%)	At. (%)	Wt. (%)	At. (%)	Wt. (%)	At. (%)	Wt. (%)	At. (%)	Wt. (%)	At. (%)	
Sample 1	25.29	35.12	1.46	1.74	3.84	4.00	65.17	57.21	2.51	1.49	1.72	0.44	100.00
Sample 2	24.88	36.08	0.69	0.86	3.55	3.86	61.46	56.35	0.98	0.61	8.44	2.25	100.00
Sample 3	23.68	36.89	1.46	1.95	7.23	8.45	46.58	45.88	2.10	1.40	18.95	5.43	100.00

### Fourier transform-infrared spectral analysis

**Figure 6:**
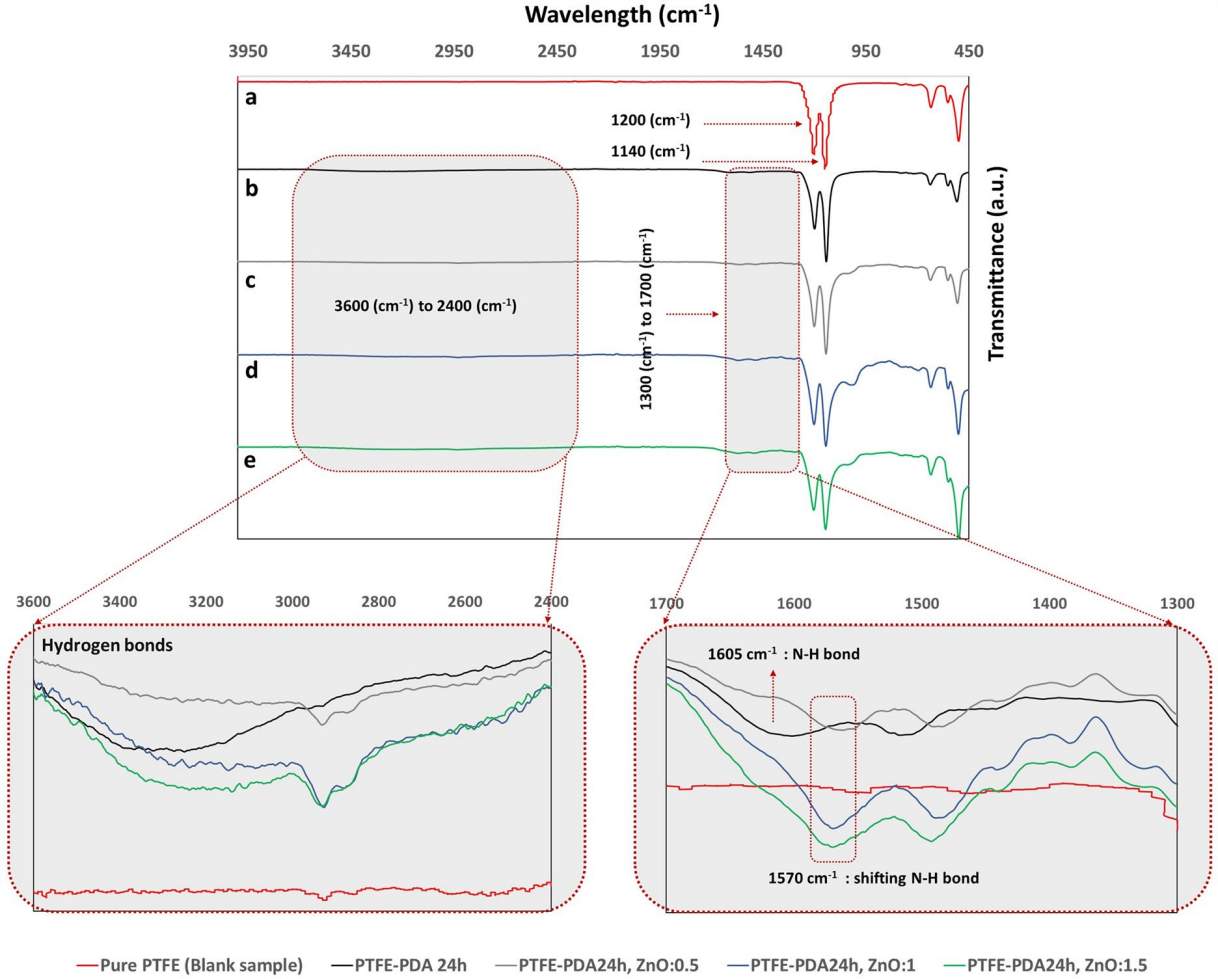
Fourier transform-infrared analysis of (**a**) Pristine PTFE (Blank), (**b**) Pdopa24@PTFE HFM, (**c**) Sample 1 (ZnO 0.5 & Pdopa 24@PTFE HFM), (**d**) Sample 2 (ZnO 1 & Pdopa 24@PTFE HFM), and (**e**) Sample 3 (ZnO 1.5 & Pdopa 24@PTFE HFM).

FT-IR analysis demonstrated the existence of both Pdopa functional groups and silane-mediated ZnO immobilization on the surface of the PTFE HFMs, with ATR-FTIR spectra before and after surface modification (Fig. 6) showing two tapered peaks attributable to the stretching vibration of C–F bonds around 1140 cm^−1^ and 1200 cm^−1^^53,54^. FT-IR spectra of the Pdopa-coated PTFE HFM showed two new peaks (Fig. 6) associated with the covalent bonds of C–H, N–H, and O–H between 1300 and 1700 cm^−1^, and hydrogen bonds of O–H and N–H between 2400 and 3600 cm^−1^^43,45,55^. A peak at 1605 cm^−1^ in the 24 h Pdopa-modified sample was attributed to the deformation vibration of the amine N–H bond, which then shifted to 1570 cm^−1^ in the samples incorporating crosslinked ZnO NPs^55^. Unlike the unmodified and other Pdopa-coated PTFE HFMs, a new broad peak appeared around 1010 cm^−1^ attributed to Si–O bond vibration^56^. These new broad peaks were representative of APTES and could only be seen in samples containing ZnO NPs. Thus, FT-IR analysis confirmed successful PTFE HFM surface treatment with Pdopa and silane-mediated ZnO NPs.

### Water contact angle and membrane porosity

**Figure 7:**
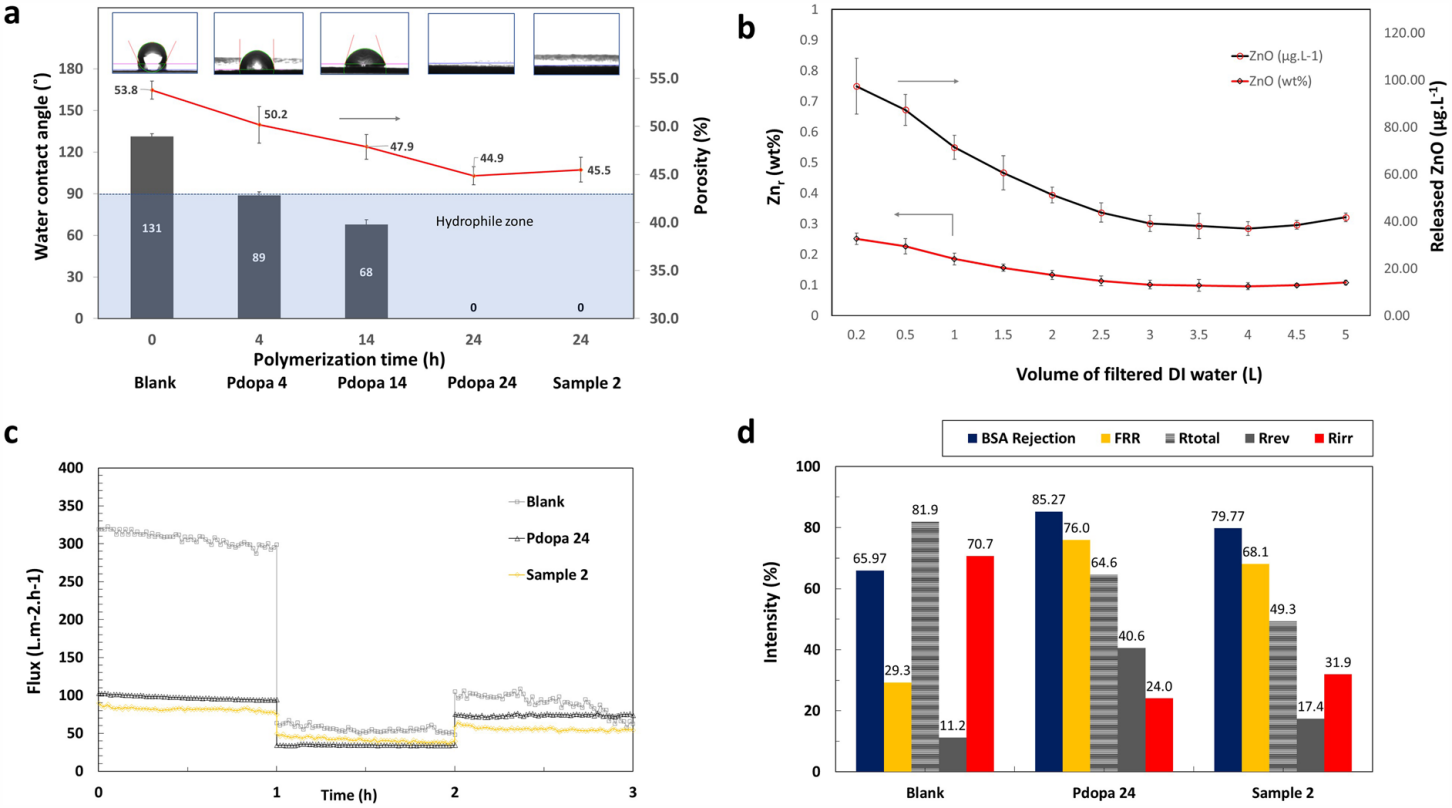
(**a**) Water contact angle and porosity analysis; (**b**) Leaching stability analysis of immobilized ZnO NPs released during the filtration procedure; (**c**) Membrane flux recovery diagrams; and (**d**) BSA rejection, FRR, Rtotal, Rrev, and Rirr values.

The water contact angle test, used to assess the effect of Pdopa modification on PTFE HFM surface characteristics, confirmed increased hydrophilicity as aerated-Pdopa reaction time increased, with the water contact angle declining from 131° to 0° after 24 h aerated polymerization (Fig. 7a). After 24 h polymerization, attachment of polar groups to the PTFE HFM surface increased the interaction between water and membrane surface, increasing its hydrophilicity to superhydrophilic levels. The water contact angle results after 24 h Pdopa treatment are in good agreement with those from SEM imaging (Figs. 4d and 5d), which confirmed uniform coverage of the whole PTFE HFM surface. Immobilization of ZnO NPs using a low concentration of APTES (2%V/V) for 1 h, followed by removal of non-reacted agents from the surface, had no negative effect on the hydrophilicity of the modified membrane surface.

In accordance with the water flux and water contact angle results, higher Pdopa deposition times led to a slight decrease (from 54 to 45%) in membrane porosity as growth of the Pdopa layer on the membrane surface constricted the membrane’s pores^34,57^, (Fig. 7a). Nevertheless, one notable advantage of utilizing the Pdopa modification technique is its inherent propensity to adhere on the surface/substrate and grow on it, rather than within the pores^19^. This characteristic explains why it did not entirely obstruct the membrane pores in comparison to other coatings/techniques. Again, incorporation of ZnO NPs onto the membrane surface had no significant impact on membrane porosity, which remained at approximately 45%.

### Stability of ZnO NPs in membranes

Following ICP-OES analysis, after initial filtration of 200 mL DI water, 97.41 µg L^−1^ of Zn was released from the membrane surface into the permeate (Fig. 7b). As the operation proceeded, the quantity of Zn released gradually declined to a relatively constant equilibrium value of around 40.00 µg L^−1^. However, considering the proportionally released Zn element of all immobilized one (i.e. 38,721.43 µg L^−1^) reveals that lower than 0.5 wt% of the immobilized ZnO NPs released from the membrane surface during the operation, confirming the overall stability of ZnO NPs on the PTFE HFM surface. Moreover, the stability evaluation of the modified coating was performed at a higher transmembrane pressure (1.0 bar) compared to the standard operating pressure for these membranes, which not exceeded usually 0.3 bar.

### Membrane flux recovery

The membrane flux recovery ratio (FRR) corresponds to its capability to recover its initial flux after filtration and cleaning cycles. A higher FRR shows a better capability of the membrane to retrieve its performance and maintain its filtration efficiency over time. A membrane with proper anti-fouling characteristics is less susceptible to fouling, which occurs when suspended particles, microorganisms, proteins, or other pollutants accumulate on the membrane surface, leading to the pores' blockage and declining the membrane's flux. However, a membrane with high anti-fouling properties can help to easily remove the fouling layer and retrieve its initial flux after cleaning or relaxation time cycle. Therefore, a higher FRR is associated with better fouling resistance and anti-fouling properties.

The percentage ability of surface-modified membranes to recover from the initial flux after BSA filtration was evaluated against a pristine membrane. The initial water flux (J_1_) of uncoated PTFE HFMs, Pdopa-coated PTFE HFMs, and samples modified with ZnO NPs was 307, 97.2, and 81.7 L m^−2^ h^−1^, respectively (Fig. 7c). The initial water flux of the Pdopa-coated membrane was lower than that for the uncoated PTFE HFM due to growth of the Pdopa layer on the membrane after 24 h polymerization, which affected the membrane pore size and porosity. Fluxes obtained during BSA filtration step for all membranes were considerably lower than those for the initial and secondary fluxes obtained during water filtration due to the adsorption and deposition of BSA molecules on the HFM surface. This observation is also in accordance with other previously reported studies^12,46^ (Fig. 7d).

While the FRR for Pdopa-modified membranes and membranes with PDA-incorporated ZnO NPs were 76 and 68%, respectively, the FRR for pristine PTFE HFMs achieved only 29% (Fig. 7d). Low FRR for pristine PTFE HFMs was presumably caused by the deposition and adsorption of BSA on the membrane surface, which results in severe membrane fouling. Consistent with earlier research^12,16,36^, the surface polymerization utilizing Pdopa to achieve a hydrophilic surface on PTFE HFMs demonstrates a significant enhancement in the flux recovery by discourage BSA attachment on the membrane surface. This consequently improves anti-fouling properties due to resist protein fouling which help easily wipe the protein molecules out of the modified membrane surface, giving them a higher FRR^12,16,36^.

Secondary flux (J_2_) values were always lower than those for the initial flux (J_1_) due to ongoing fouling of the membranes (Fig. 7c). The initial membrane flux loss corresponds to total membrane fouling (R_total_), which comprises both reversible (R_rev_) and irreversible (R_irr_) fouling^42^, i.e. fouling removed by physical water cleaning and fouling requiring chemical agents for cleaning^46^. Surface modification would be deemed favorable, therefore, if obtained a proportionally lower R_total_, R_irr_ and a higher percentage of R_rev_^46^. In our study, the R_total_ value for pristine PTFE HFM (81.9%) was higher than that for Pdopa-modified membranes (64.6%) and Pdopa incorporating ZnO NPs (49.3%), indicating a greater tendency for fouling. In other words, the modified membranes showed improved anti-fouling resistivity. Furthermore, the membrane rejection ratio of BSA also improved from 66.0% for the pristine membrane to 85.3% for membrane after surface modification with Pdopa and to 79.8% after surface modification with Pdopa incorporating ZnO NPs (Fig. 7d).

To summarise, Pdopa surface modification improved PTFE HFM anti-fouling properties by providing a barrier layer on the surface that moderated attractive sites for protein molecules and facilitated the removal of adsorbed BSA molecules by water cleaning^12^. The modified membranes had an improved rejection ratio against BSA filtration. While the anti-fouling properties of Pdopa-modified membranes were higher than those containing ZnO NPs, ZnO NP-modified membranes still showed improved FRR levels over pristine PTFE HFMs which corresponds to the anti-fouling propensity of the modified membrane.

### Membrane antimicrobial properties

**Figure 8:**
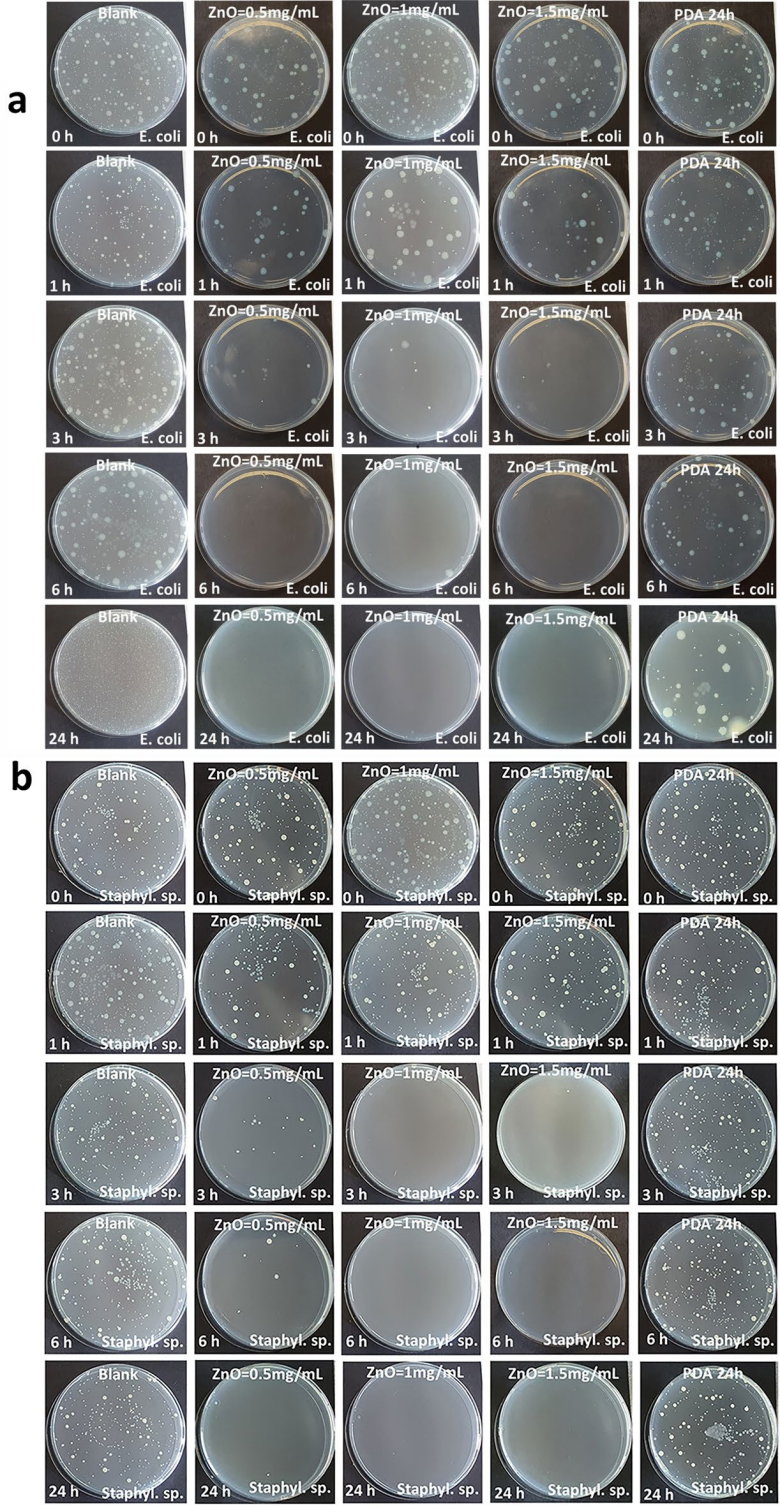
Dynamic antimicrobial analysis activity against: (**a**) *Escherichia coli*, and (**b**) *Staphylococcus* sp.

Evaluation of CFUs for pristine PTFE HFMs indicated no inhibition activity against either *Staphylococcus* sp. or *E. coli* bacteria, indicating susceptibility to biofouling during operation in a bioreactors (Fig. 8a,b). On contrast to previous studies reporting on Pdopa imparting antibacterial properties^26,27^, our Pdopa-modified membranes showed no favorable antimicrobial activity against either strain of bacteria (Fig. 8a,b). It can be explained by additional modification of the Pdopa coating with ZnO NPs, which is being frequently used as a non-toxic inorganic antimicrobial agent^31,33^ for immobilizing HFMs. The subsequent microbial assessment confirmed improved resistivity against bacteria for membranes modified with ZnO NPs (Samples 1, 2, 3; Table 2), even at low contact times (Fig. 8a,b).

**Figure 9:**
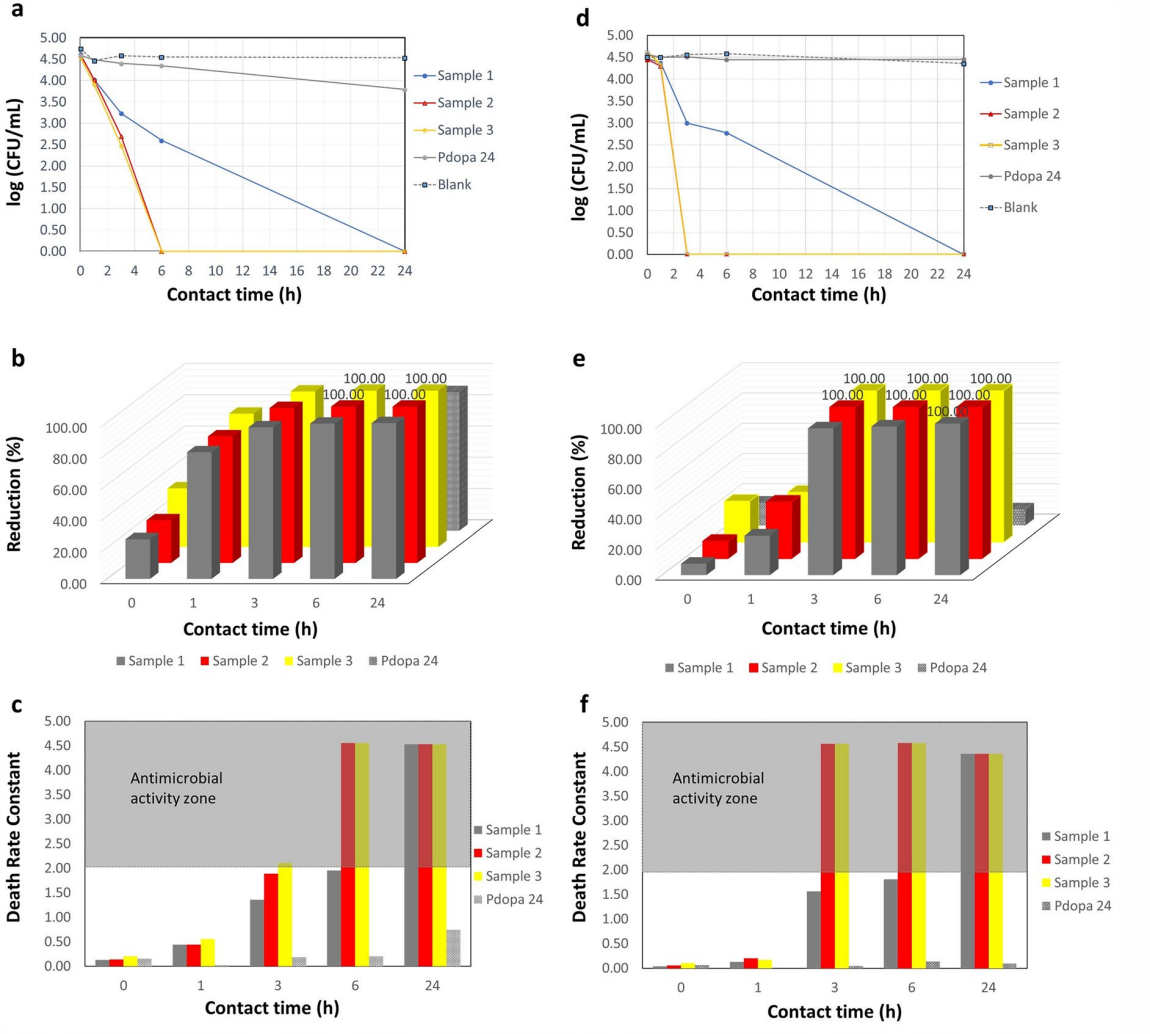
Antimicrobial activity of pristine and surface-modified PTFE HFMs: total number of CFU (logarithmic scale) against (**a**) *Escherichia coli* and (**d**) *Staphylococcus* sp.; reduction value (RV) against (**b**) *Escherichia coli* and (**e**) *Staphylococcus* sp.; and reduction percentage (R%) against (**c**) *Escherichia coli* and (**f**) *Staphylococcus* sp.

All membranes containing ZnO NPs showed antimicrobial activity immediately after inoculation with the bacterial solutions (Fig. 9a,d). Samples 2 and 3, which had higher ZnO NP concentrations, showed 100% reduction after 6 and 3 h contact time against *E. coli* and *Staphylococcus* spp., respectively. In contrast, it took 24 h for Sample 1 (containing 0.5 mg mL^−1^ ZnO NPs) to reach 99% bacterial reduction against *E. coli* and 100% against Staphylococcus sp. (Fig. 9b,e).

Constant reduction values (values > 2 indicating effective antimicrobial action^58^) also confirmed the effectiveness of ZnO NPs at improving antimicrobial resistivity, with Samples 2 and 3 both having bacterial reduction values of > 2 after only 6 h and 3 h contact, respectively, against *E. coli*, and 3 h for both against *Staphylococcus* sp. (Fig. 9c,f). Sample 1 also showed effective antimicrobial resistivity, but only after 24 h inoculation with both bacterial strains. After 24 h inoculation, all three samples of 1, 2, and 3 achieved reduction values of 4.53 against *E. coli* and 4.36 against *Staphylococcus* sp. bacteria. Furthermore, reduction values confirmed the lack of antimicrobial activity shown by Pdopa-modified membranes, with the highest reduction values after 24 h contact time being as low as 0.74 and 0.1 after inoculation with *E. coli* and *Staphylococcus* sp., respectively. The anti-(bio)fouling performance of membranes examined in this study was also compared with other studies employing Pdopa for surface modification of PTFE (flat-sheet and hollowfiber) membranes (Table 4). There have been only few studies on superhydrophilic PTFE HFM showing anti-(bio)fouling properties for gram-positive and gram-negative bacteria. Therefore, modification of PTFE by Pdopa is a promising option for new membranes suitable for use in (waste)water treatment applications.

**Table 4:** Comparison among recent studies on hydrophilized Pdopa utilized surface modification of PTFE (hollow fiber and flat sheet) membranes.

Membrane	Type	Modification layer	Hydrophilicity		Fouling resistivity		Application	References
		Layer	Result	WCA* (°)	Anti-fouling	Anti-(bio)fouling		
PTFE	Hollow fiber	Pdopa–ZnO NPs	Superhydrophile	0	Flux recovery by Pdopa layer > 76% flux recovery by final layer > 68%	100% Against *E. coli* 100% against *Staphylococcus* sp.	Wastewater treatment	Present study
PTFE	Hollow fiber	Pdopa/polyamide	Hydrophile	85.8°	Improve resistivity against aprotic solvents solvent rejections > 90%, but no report on flux recovery	No data	Organic solvent nanofiltration	^59^
PTFE	Hollow fiber	Pdopa/polyethyleneimine (PEI)	Superhydrophile	0	Flux improvement, but no report on flux recovery	No data	TiO_2_ suspension separation	^11^
PTFE	Flat sheet	Pdopa	Superhydrophile	0	Flux recovery ≈ 90%	No data	Oil/water separation	^60^
PTFE	Flat sheet	Pdopa	Hydrophobe	94.3°	73.8% FRR (against NaCl solution 3.5%)	No data	Water desalination	^61^
PTFE	Flat sheet	(P(DMAEMA-co-TlaAm))**/Pdopa	Ultra-hydrophile	13°	Flux recovery by Pdopa layer > 90% flux recovery by Ptla layer > 95%	86% Against *E. coli* 99% against *S. aureus*	Oil/water separation	^62^
PTFE	Flat sheet	Pdopa/Fe_3_O_4_ NPs/Pdopa	Hydrophile	25°–30°	Membrane flux stability improvement (0.6–0.7 LMH), but no report on flux recovery after fouling	No data	Water desalination	^63^
PTFE	Flat sheet	Pdopa/Ag NPs/Pdopa	Hydrophile	62°–56°	Flux improvement, but no report on flux recovery	No data	Water desalination	^64^

*WCA water contact angle; **(P(DMAEMA-co-TlaAm)) = synthesized polymer containing thiolactone and glucosamine copolymer with tertiary amine moieties.

## Conclusion

In the present study, for the first time, PTFE HFMs were subjected to air-stimulated surface modification based on the polymerization of Pdopa incorporating ZnO NPs to induce superhydrophilic surface modification. Multi-scale testing indicated that the optimum modified coating was prepared by 24 h of air-stimulated polymerization of Pdopa (2.0 mg mL^−1^ in pH = 8.5 Tris-buffer solution), followed by immobilization of ZnO NPs (1.0 mg mL^−1^ by 2%v/v APTES in EtOH). Subsequent morphological characterization confirmed the positive impact of diffused aeration for accelerating the homogeneous growth of a Pdopa layer on the PTFE membrane surface, forming superhydrophilic surface due to uniform Pdopa coverage, confirmed by water contact angle values. Immobilization of ZnO NPs was determined by FT-IR spectra and EDX analysis, and ICP analysis confirmed coating stability during membrane filtration. Functional improvement after superhydrophilic modification was assessed through protein filtration and evaluation of the antimicrobial activity. Overall, flux recoveries increased to 76% after 24 h coating with Pdopa, and 68% by the ZnO NPs incorporation. Furthermore, the surface-modified membranes showed a reduced tendency for fouling (64.6% for 24 h Pdopa coating and 49.3% for ZnO NPs immobilization) against pristine membranes (81.9%). A bacterial reduction rate of 100% was recorded for the optimally modified membrane, even after short inoculation times of 6 h and 3 h against *E. coli* and *Staphylococcus* sp., respectively. Consequently, our results confirm that air-stimulated membrane modification based on Pdopa coating and incorporation of ZnO NPs as an antimicrobial agent provides advanced fouling resistance and sustainable superhydrophilic surface properties to PTFE HFMs, allowing for their potential use in (waste)water treatment processes.

## Data Availability

The data for this study are available from the corresponding author upon on a reasonable request.
